# Does the use of ketamine or nitroglycerin as an adjuvant to lidocaine improve the quality of intravenous regional anesthesia?

**DOI:** 10.4103/1658-354X.65122

**Published:** 2010

**Authors:** Khaled Fawzy Elmetwaly, Nasr Abdelmohsen Hegazy, Abdelkhalek Abdelmonem Aboelseoud, Ahmad Abdullah Alshaer

**Affiliations:** *Assistant Professor of Anesthesia, College of Medicine, Ain Shames University, Cairo, Egypt*; 1 *Assistant Professor of Cardiac Anesthesia, King Fahd Cardiac Centre, King Saud University, Riyadh, KSA*

**Keywords:** *Intravenous regional anesthesia*, *NMDA receptor antagonist*, *ketamine*, *nitroglycerin*, *tourniquet pain*

## Abstract

**Aims:**

To compare and evaluate the effect of adding ketamine or nitroglycerin (NTG) as adjuncts to lidocaine for intravenous regional anesthesia (IVRA) on intraoperative and postoperative analgesia, sensorial and motor block onset times, and tourniquet pain.

**Settings and Design:**

A prospective, randomized, double-blind study was carried out.

**Materials and Methods:**

Seventy-five patients undergoing hand surgery were divided into three groups as follows: control group receiving lidocaine 2%, LK group receiving lidocaine 2% with ketamine, and LN group administered lidocaine 2% with NTG. Sensory and motor blocks' onset and recovery times were recorded. Visual analog scale (VAS) for tourniquet pain was measured after tourniquet application and it was also used to measure postoperative pain. Analgesic consumption for tourniquet pain and postoperatively were recorded.

**Results:**

Sensory block onset times were shorter in the LK (4.4 ± 1.2 minutes) and LN (3.5 ± 0.9 minutes) groups compared with the control group (6.5 ± 1.1 minute) (*P* < 0.0001) and motor block onset times were shorter in the LK (7.3 ± 1.6 minutes) and LN (3.6 ± 1.2 minutes) groups compared with the control group (10.2 ± 1.5 minutes) (*P*< 0.0001). Sensory recovery time prolonged in the LK (6.7 ± 1.3 minutes) and LN (6.9 ± 1.1 minutes) groups compared with the control group (5.3 ± 1.4 minutes) (*P* = 0.0006 and < 0.0001, respectively). Motor recovery time prolonged in the LK (8.4 ± 1.4 minutes) and LN (7.9 ± 1.1 minutes) groups compared with the control group (7.1 ± 1.3 minutes) (*P* = 0.0014 and 0.023, respectively). The sensory and motor block onset times were also shorter in LN group than in the LK group (3.5 ± 0.9 versus 4.4 ± 1.2 minutes, *P*=0.004; and 3.6 ± 1.2 versus 7.3 ± 1.6 minutes, *P* < 0.0001, respectively). The amount of fentanyl required for tourniquet pain was less in adjuvant groups when compared with control group. It was 13.6 ± 27.9 and 27.6 ± 34.9 *µ*g in LK group and LN groups, respectively, versus 54.8 ± 28 *µ*g in the control group. VAS scores of tourniquet pain were higher at 10, 20, 30, 40 minutes in the control group compared with the other study groups (P < 0.0001). It was also higher in LN group compared with LK group at 30 and 40 minutes (*P* < 0.001). Postoperative VAS scores were higher for the first 4 h in control group compared with the other study groups (*P*< 0.0001).

**Conclusions:**

The adjuvant drugs (ketamine or NTG) when added to lidocaine in IVRA were effective in improving the overall quality of anesthesia, reducing tourniquet pain, increasing tourniquet tolerance and improving the postoperative analgesia in comparison to the control group. Ketamine as an adjuvant produced better tolerance to tourniquet than the other groups. NTG as an adjuvant produced faster onset of sensory and motor blockades in comparison to other groups.

## INTRODUCTION

Intravenous regional anesthesia (IVRA) was first described by August K.G. Bier in 1908 for anesthesia of the hand and forearm.[1] The technique regained popularity in the 1960s when Holmes used lidocaine.[2] IVRA is simple to administer, reliable, and cost-effective.[3] It is ideal for short operative procedures on the extremities performed on an ambulatory basis. Disadvantages include concerns about local anesthetic (LA) toxicity, slow onset, poor muscle relaxation, tourniquet pain, and minimal postoperative pain relief.[4] The ideal IVRA solution should have the following features: rapid onset, reduced dose of LA, reduced tourniquet pain, and prolonged post-deflation analgesia. At present, this may only be achieved by the addition of adjuncts to LA. Several adjuncts have been used including narcotics, nonsteroidal anti-inflammatory drugs (NSAIDS), muscle relaxants, α2 agonists and neostigmine.[5]

Ketamine, a phenyl-piperidine derivative, was first synthesized in the early 1960s and marketed as an intravenous anesthetic at the beginning of the seventies. At subanesthetic (i.e., low) doses, ketamine exerts a noncompetitive blockade of *N*-methyl-D-aspartate (NMDA) receptors. NMDA receptors play a major role in synaptic plasticity and are specifically implicated in central nervous system (CNS) facilitation of pain processing. Therefore, NMDA receptor antagonists have been implicated in perioperative pain management as they modulate central sensitization induced both by the incision and tissue damage.[6] Nitroglycerin (NTG), a nitric oxide generator, has been demonstrated to enhance the analgesic action of many drugs in acute and chronic pain conditions. It enhances the analgesia of oral morphine in chronic cancer pain,[7] and the antinociception from spinal administration of either sufentanil[8] or neostigmine,[9] and in acute postoperative pain. Also, it was noticed that the addition of NTG to lidocaine for IVRA improves postoperative analgesia.[10]

This study was designed to compare and evaluate the effect of adding ketamine or NTG as adjuncts to lidocaine for IVRA, on intraoperative and postoperative analgesia, sensorial and motor block onset times, and tourniquet pain.

## MATERIALS AND METHODS

After approval of the local ethics committee and securing informed patient consent from 75 patients, ASA physical status I–II patients, aged between 20 and 50 years, who were undergoing elective hand or forearm surgery (i.e., carpal tunnel, trigger finger, and tendon release) were included in this prospective, randomized, double-blind study. The patients were randomly allocated to one of three groups; each group consisted of 25 patients. The sample size of this study was chosen after reviewing many randomized control studies concerning with the same subject and had a sample size ranging between 20 and 40 patients.[5] Randomization was performed using a closed envelope method. Patients with Reynaud's disease, sickle cell anemia, or history of drug allergy were excluded from the study.

After establishing noninvasive arterial blood pressure, electrocardiogram, and peripheral oxygen saturation monitoring (Drager Cicero EM, Germany), two venous cannulae were inserted: one in a vein on the dorsum of the operative hand (22-gauge) and the other in the opposite hand for crystalloid infusion. The operative arm was elevated for 2 minutes, and using an Esmarch bandage, the venous capacitance of the arm was emptied. Then, a double-pneumatic tourniquet was applied. The selection of tourniquets is closely related to the diameter and length of the arm. They were 40% bigger than the diameter of the arm and 5–6 cm wide each. The proximal tourniquet was inflated to a pressure of 250 mmHg. Circulatory isolation of the arm was confirmed by inspection, lack of radial pulse, and failure of pulse oximetry tracing of the ipsilateral index finger. After the Esmarch bandage was released, patients in group L (lidocaine or control group *n* = 25) received 2% lidocaine (lidocaine 2%, B. Braun, Germany) 3 mg/kg (maximum 200 mg) for IVRA, patients in group LK (lidocaine ketamine group *n* = 25) received 2% lidocaine 3 mg/kg (maximum 200 mg) plus 0.1 mg/kg ketamine, (Ketam, Hekma pharmaceuticals, Jordan), and those in group LN (lidocaine NTG group *n* = 25) received 2% lidocaine 3 mg/kg (maximum 200 mg) for IVRA and 200 *µ*g NTG (Solinitrina, Almirall Laboratories, Espane). The drugs were prepared in the pharmacy, and in all groups 0.9% NaCl was added to make up a total volume of 40 mL. The IVRA solution was administered over 60s by an anesthesiologist who was blinded to the drug being administered. After anesthesia was achieved, the distal tourniquet was inflated to 250 mmHg pressure, and the proximal tourniquet was deflated. The tourniquet and operative times were recorded.

Sensory block was assessed by a pinprick test performed with a 22-gauge short-beveled needle every 30s. The patient's response was evaluated in the dermatomal sensory distribution of the ulnar, median, and radial nerves. Sensory block onset time was noted as the time from injection of the study drug until sensory block in all dermatomes. Motor function was assessed every minute by asking the patient to flex and extend the wrist and fingers; complete motor block was noted when no voluntary movement was possible. Motor block onset time was the time elapsed from injection of study drug to complete motor block.

The tourniquet was not deflated before 30 minutes and was not inflated for more than 1.5h. At the end of surgery, tourniquet deflation was performed using the cyclic deflation technique. Sensory recovery time (time elapsed after tourniquet deflation to recovery of pain sensation in all dermatomes determined by pinprick test) was noted. Motor block recovery time (the time after tourniquet deflation to movement of fingers) was noted.

Arterial blood pressure, heart rate, and peripheral oxygen saturation were recorded preoperatively (baseline), 1 minute after inflation of the tourniquet, and every 5 minutes after administration of drug till tourniquet release, and after transfer to the recovery room at 5, 30, and 60 minutes. During the operation, tourniquet pain and after surgery pain at the operative site were assessed by visual analog scale (VAS) (0 = no pain and 10 = worst pain imaginable).

Intraoperatively, boluses of 1 *µ*g/kg fentanyl were supplied for tourniquet pain treatment at any necessary time (when VAS >3), and the amount of fentanyl consumption was recorded. Postoperatively, the patients were instructed to receive 16 mg IV lornoxicam once daily when VAS was >3, and total amounts of lornoxicam (Xefo, Jazera pharmaceutical industries, KSA) administered to each group were recorded.

At the end of the operation, the blind anesthesiologist to the test group was asked to qualify the operative conditions according to following numeric scale: 4 = excellent (no complaint from patient), 3 = good (minor complaint with no need for supplemental analgesics), 2 = moderate (complaint which required supplemental analgesic), and 1 = unsuccessful (patient given general anesthesia). The surgeon, who was not aware of the given medication, was inquired to qualify the operative situation consistent with the following numeric scale: 1 = unsuccessful, 2 = poor, 3 = acceptable, and 4 = perfect.

Through the study period, any local or systemic complications including nausea, vomiting, skin rash, tachycardia, bradycardia, hypotension, hypertension, headache, dizziness, tinnitus, hypoxemia, sedation, respiratory depression, bradypnea, tachypnea, hallucination, nystagmus, or any other side effects were noted. These measurements were recorded by an anesthesiology resident who did not know which medication was administered. Measurements in all patients were performed by the same person.

The statistical evaluation was performed using SPSS version 11.0. Independent samples' *t*-test was used for evaluation of the demographic data, intraoperative or postoperative hemodynamic data, the time of the onset or recovery of sensory and motor blocks, the duration of the operation and tourniquet, and intraoperative or postoperative analgesic use. The Kruskal–Wallis test was used for intraoperative and postoperative VAS and the quality of anesthesia. Operation type, the number of patients who required analgesia for tourniquet pain, and complications were compared using the chi-square test. A *P* value of < 0.05 was considered significant.

## RESULTS

Demographic data of the groups were comparable for mean age, weight, and sex ratio. There was no statistical difference in the types of surgical procedure, duration of surgery, and tourniquet time [Table 1].

**Table 1:** Demographic data, tourniquet time, operative time, and types of surgery

Variable	Control L group (*n* = 25)	Ketamine LK group (*n* = 25)	NTG LN group (*n* = 25)
Age (years)	35 (25–45)	40 (27–50)	38 (20–50)
Sex (M/F)	13/12	10/15	11/14
Weight (kg)	78 ± 18	71 ± 17	75 ± 16
Tourniquet time (minutes)	50 ± 6	52 ± 8	49 ± 7
Operation time (minutes)	43 ± 7	46 ± 9	44 ± 8
Types of surgery (carpal tunnel/trigger finger/tendon release) (*n*)	10/7/8	11/5/9	9/6/10

Values are expressed in mean (SD) and ratio

**Figure 1:**
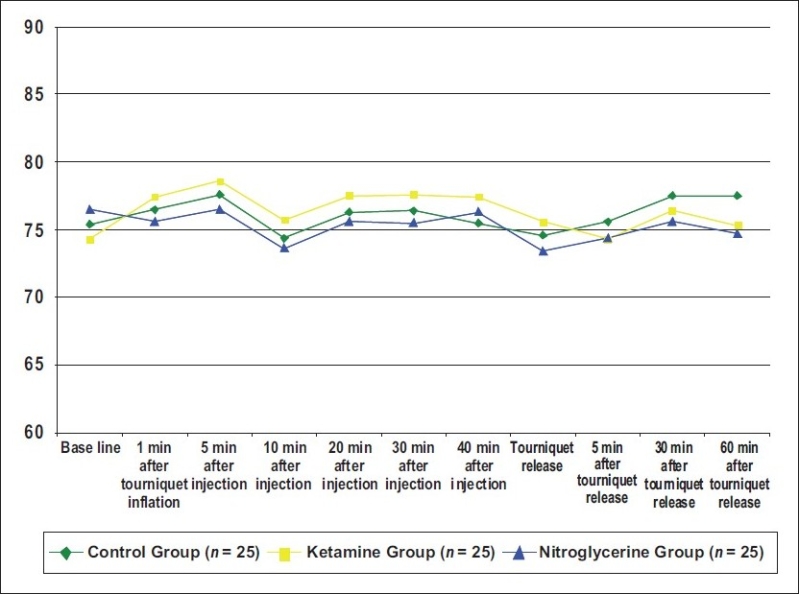
Mean values of the heart rate of the three groups

**Figure 2:**
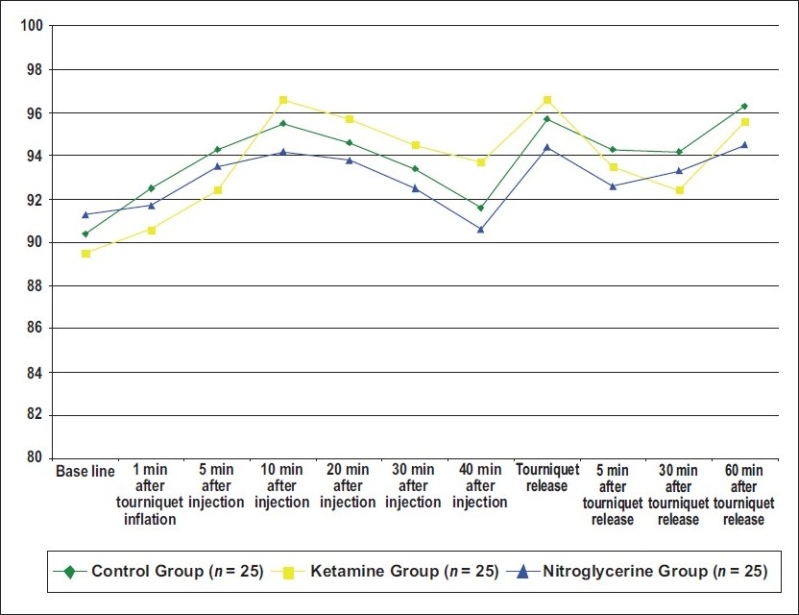
Mean values of the mean blood pressure of the three groups

**Figure 3:**
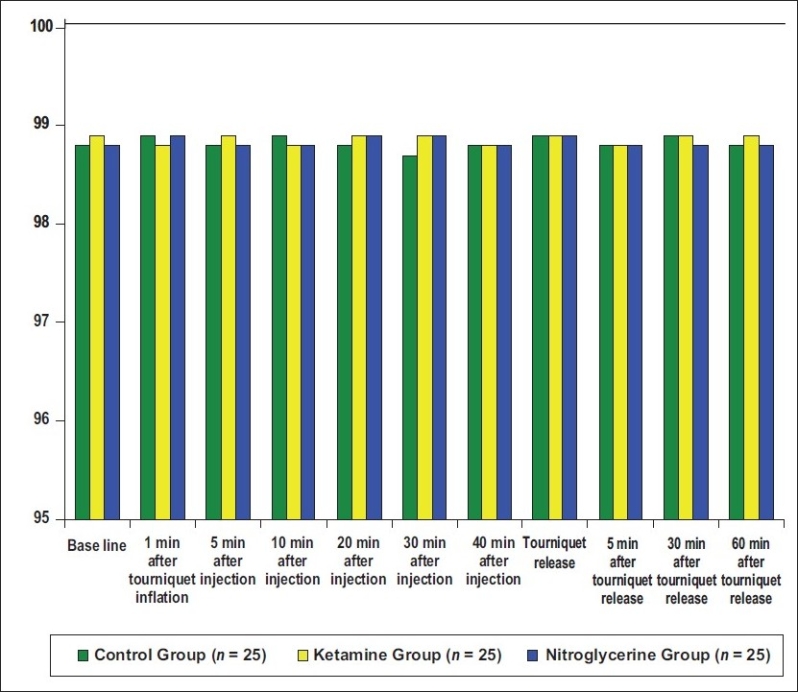
Median values of the oxygen saturation of the three groups

There was also no statistical difference between groups when compared for heart rate, mean arterial pressure, and saturation at any preoperative, intraoperative, and postoperative times (*P* > 0.05) [Figures 1‐3].

Sensory block onset times were shorter in the LK (4.4 ± 1.2 minutes) and LN (3.5 ± 0.9 minutes) groups compared with the control group (6.5 ± 1.1 minutes) (*P* < 0.0001), and motor block onset times were shorter in the LK (7.3 ± 1.6 minutes) and LN (3.6 ± 1.2 minutes) groups compared with the control group (10.2 ± 1.5 minutes) (*P* < 0.0001). Sensory recovery time prolonged in the LK (6.7 ± 1.3 minutes) and LN (6.9 ± 1.1 minutes) groups compared with the control group (5.3 ± 1.4 minutes) (*P* = 0.0006 and < 0.0001, respectively).

**Figure 4:**
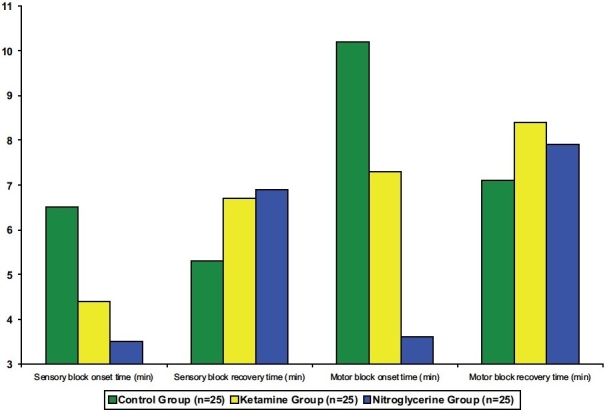
Onset and recovery times of sensory and motor blocks

Motor recovery time prolonged in the LK (8.4 ± 1.4 minutes) and LN (7.9 ± 1.1 minutes) groups compared with the control group (7.1 ± 1.3 minutes) (*P* = 0.0014 and 0.023, respectively). The sensory and motor block onset times were also shorter in LN group than in LK group (3.5 ± 0.9 versus 4.4 ± 1.2 minutes, *P* = 0.004 and 3.6 ± 1.2 versus 7.3 ± 1.6 minutes, *P* < 0.0001, respectively). There was no statistical difference in the sensory and motor recovery times between the adjuvant groups [Figure 4].

No patient suffered from incisional pain during intraoperative period in any of the study groups. The amount of fentanyl required for tourniquet pain was less in adjuvant groups when compared with the control group. It was 13.6 ± 27.9 and 27.6 ± 34.9 *µ*g in the LK and LN groups, respectively, versus 54.8 ± 28 *µ*g in the control group (*P* < 0.0001 and 0.004, respectively).

Supplemental fentanyl was required for tourniquet pain in 20 patients in control group and 10 patients in LK group, but for only 5 patients in LN group. The first time for fentanyl requirement for tourniquet pain was prolonged in the LN group when compared with the control group (28.2 ± 7.4 minutes versus 15.8 ± 6.3 minutes, *P* < 0.0001), whereas in the LK group it was 38.3 ± 8.5 minutes, making it statistically significant than both the control and even the LN groups with *P* < 0.0001 [Table 2]. Lornoxicam consumption was lower in the adjuvant groups compared with the control group. It was 6.4 ± 8.0 and 3.2 ± 6.5 mg in the LK and LN groups, respectively, versus 12.8 ± 6.5 mg in the control group (*P* = 0.003 and < 0.0001, respectively) [Table 2].

**Table 2:** The amount of intraoperative analgesia, time of fentanyl requirement for tourniquet pain, the number of patients who required analgesia for tourniquet pain, and the amount of postoperative analgesics

Variable	Control L group (*n* = 25)	Ketamine LK group (*n* = 25)	NTG LN group (n = 25)
Amount of intraoperative analgesic (fentanyl in micrograms)	54.8 ± 28.7	13.6 ± 27.9*	27.6 ± 34.9*
Time of fentanyl requirement for tourniquet pain (minutes)	15.8 ± 6.3	38.3 ± 8.5*	28.2 ± 7.4**
Number of patients who required analgesia for tourniquet pain	20/25	5/25*	10/25*
Amount of postoperative analgesic (lornoxicam in milligrams)	12.8 ± 6.5	6.4 ± 8.0*	3.2 ± 6.5*

Values are expressed in mean (SD) and ratio;

* *P* < 0.05 in comparison with the control group.

** *P* < 0.05 between ketamine and NTG groups.

VAS scores of tourniquet pain were higher at 10, 20, 30, 40 minutes in control group compared with the adjuvant groups (*P* < 0.0001). It was also higher in LN group compared with LK group at 30 and 40 minutes (*P* < 0.001). Postoperative VAS scores were statistically highly significant for the first 4 h in control group compared with the adjuvant groups (*P* < 0.0001) [Table 3].

**Table 3:** Intraoperative tourniquet pain and postoperative VAS scores

Variable	Control L group (*n* = 25)	Ketamine LK group (*n* = 25)	NTG LN group (*n* = 25)
Before tourniquet	0	0	0
5 minutes after tourniquet inflation	1 (0–1)	1 (0–1)	1 (0–1)
10 minutes after tourniquet inflation	2 (0–3)	1 (0–2)*	1 (0–2)*
20 minutes after tourniquet inflation	2 (0–3)	1 (0–2)*	1 (0–3)*
30 minutes after tourniquet inflation	3 (1–4)	1 (1–2)*	2 (1–3)**
40 minutes after tourniquet inflation	3 (2–5)	2 (1–3)*	3 (2–4)**
Tourniquet release	3 (2–5)	2 (2–3)*	3 (2–4)*
30 minutes after tourniquet release	3 (2–5)	2 (2–5)*	2 (2–4)*
2 h after tourniquet release	4 (3–5)	3 (3–5)*	3 (2–5)*
4 h after tourniquet release	4 (3–5)	3 (3–5)*	3 (2–5)*

Values are expressed as median (range);

* *P* < 0.05 in comparison with control group.

** *P* < 0.05 between ketamine and NTG groups.

Anesthesia quality, determined by the anesthetist and the surgeon, was better in adjuvant groups than in the control group (*P* < 0.0001) [Table 4].

**Table 4:** Quality of anesthesia assessed by anesthetist and surgeons

Variable	Control L group (*n* = 25)	Ketamine LK group (*n* = 25)	NTG LN group (*n* = 25)
Quality of anesthesia (anesthetist)	3 (2–4)	4 (3–4)*	4 (3–4)*
Quality of anesthesia (surgeon)	2 (2–3)	4 (3–4)*	3 (3–4)*

Values are shown as median (range);

* *P* < 0.05 in comparison with the control group.

There was no patient in the three groups converted to general anesthesia because of insufficient anesthesia and no other side effects were seen.

## DISCUSSION

The results of our study revealed that the addition of ketamine or NTG to lidocaine for IVRA improves the speed of onset and the quality of anesthesia, decreases tourniquet pain and intraoperative and postoperative analgesic consumption, lengthens the time to the first patient's demand for lornoxicam administration after surgery and does not cause significant side effects. However, ketamine is superior in decreasing the tourniquet pain than NTG, whereas NTG provides faster sensory and motor onset times.

The injected LA diffuses into the small veins surrounding the nerves and then into the vasa nervorum and capillary plexus of the nerves, leading to a core-to-mantle (centrifugal) conduction block in the nerves involved. It then spreads around the small nerves in the skin, blocking their conduction.[11]

Pain is detected by two different types of peripheral nociceptor neurons: C-fiber nociceptors with slowly conducting unmyelinated axons and A-delta nociceptors with thinly myelinated axons. Surgical trauma results in the release of intracellular contents from damaged and inflammatory cells leading to nociceptors' sensitization, discharge spontaneously, and produce ongoing pain. Prolonged firing of C-fiber nociceptors causes release of glutamate which acts on NMDA receptors in the spinal cord. This creates a state of “wind-up” phenomenon that leads to a hyperactive nociceptive system and increases the magnitude and duration of neurogenic responses to pain, even after the initial peripheral input is stopped.[12] So, activation of NMDA receptors causes the spinal cord neuron to become more responsive to all of its inputs, resulting in central sensitization.[13] Ketamine is a noncompetitive antagonist of NMDA receptor, which can inhibit the induction of central sensitization owing to peripheral nociceptive stimulation and eliminate hypersensitivity.[14]

Ketamine also has LA qualities which have been studied as a sole agent for IVRA. Durrani *et al*.[15] found that the use of 0.3% ketamine for regional anesthesia of the upper extremity was adequate for complete sympathetic, sensory, and motor blockades. Also, Deshpande and his colleagues[16] reported similar findings. However, unpleasant psychotomimetic effects after release of the tourniquet limit its usefulness. Kaul *et al*.[17] compared ketamine (0.5%) 3 mg/kg with lignocaine (0.5%) 3 mg/kg. The onset of analgesia and motor blockade was similar in both the study groups. However, duration of analgesia after release of tourniquet was longer with ketamine. Also, the quality of analgesia was superior in the ketamine group, but all patients in the ketamine group suffered from disorientation and hallucinations. For these side effects, ketamine is not a suitable drug as a sole agent for IVRA. When ketamine was used as an adjunct in combination with LAs, it has been shown to be very effective in reducing the incidence of tourniquet pain. When ketamine is used in IVRA for this purpose, the recommended dose is 0.1 mg/kg and there are no CNS symptoms when used in this dosage.[18]

NTG exerts its analgesic effect as it is metabolized to nitric oxide (NO) in the cell.[19] NO causes an increase in the intracellular concentration of cyclic guanosine monophosphate, which produces pain modulation in the central and peripheral nervous systems.[20] NO generators also induce anti-inflammatory effects and analgesia by blocking hyperalgesia and the neurogenic component of inflammatory edema by topical application.[21]

Several studies on NTG have demonstrated its analgesic effect in acute and chronic pain conditions. Berrazueta *et al*.[22] proved the analgesic action of transdermal glycerylnitrate in the treatment of infusion-related thrombophlebitis. Lauretti *et al*.[8,9] documented that transdermal NTG enhanced the postoperative analgesic effect of spinal sufentanil and neostigmine. In cancer pain, transdermal NTG delayed morphine tolerance and decreased the frequency of adverse effects related to large doses of opioid.[13] Also, NTG has been studied as an adjuvant to IVRA. Abbasivash *et al*.[23] studied the effect of adding NTG to lidocaine in IVRA where it shortened the onset times of sensory and motor block and decreased the tourniquet and postoperative pains, without any side effect. Sen *et al*.[10] studied the addition of NTG to lidocaine in IVRA where it shortened sensory and motor block onset times, prolonged sensory and motor block recovery times, and improved tourniquet pain while prolonging the time for the first analgesic requirement and decreasing the total amount of postoperative analgesic requirement without side effects.

Although both ketamine and NGT have well-known hemodynamic effects (hypertension and tachycardia for the former and hypotension and reflex tachycardia for the latter), they fail to show any of these effects when given as adjuvants in IVRA. This can be due to the fact that the tourniquet was not deflated before 30 minutes and the tourniquet deflation was performed by the cyclic deflation technique at the end of surgery. In addition, NGT has a very short half-life (1–4 minutes),[19] whereas intravenous ketamine causes rise in the blood pressure over 3–5 minutes and then returns to normal, 10–20 minutes after injection.[24]

The rapid onset of sensory and motor blocks in NTG group than ketamine group could be explained by the direct strong vasodilator effect that promotes distribution of lidocaine to nerves.

In our study, ketamine was superior to NTG in limiting the onset and magnitude of the tourniquet pain, as evident in the more prolonged time for first fentanyl requirement for control of pain (38.3 ± 8.5 minutes for LK group versus 28.2 ± 7.4 minutes for LN group) and the lower VAS score at 30 and 40 minutes intraoperatively. This result was similar with the one got in a study done by Viscomi *et al*.[25] who demonstrated ketamine to be a valuable adjuvant in lidocaine-based IVRA.

Tourniquet pain, which is described as a dull and aching pain sensation, is a well-known limitation of IVRA. Skin compression,[26] tourniquet size,[27] inflation pressure,[28] and adjuvants in the LA solution[29] have been implicated as factors involved in tourniquet pain. Tourniquet pain is thought to be mediated by impulse propagation via small, unmyelinated, slow-conducting C fibers.[30] In addition to spinal cord NMDA receptors, NMDA receptors have also been identified on peripheral unmyelinated sensory axons. This can explain why ketamine as an NMDA receptor antagonist was superior for attenuating the tourniquet pain than NTG.

We used lornoxicam for postoperative pain as it is a new NSAID of the oxicam class with analgesic, anti-inflammatory, and antipyretic properties, and is available in parenteral form.[31] It is rapidly eliminated, having a short plasma elimination half-life of 3–5h, which suggests its suitability for acute use in the postoperative period.[32] Although both ketamine and NTG were effective in reducing postoperative pain compared with the control group, as evident in lower VAS scores and lower analgesic consumption postoperatively, our study time was short (4 h). We still need to compare between different adjuvants to discover the ideal one especially for the prolonged post-deflation analgesia.
